# Association between depression, glycaemic control and the prevalence of diabetic retinopathy in a diabetic population in Cameroon

**DOI:** 10.4102/sajpsychiatry.v23i0.983

**Published:** 2017-06-01

**Authors:** Kirsty K. Hall, Joelle Tambekou, Linda Penn, Alioune Camara, Naby M. Balde, Eugene Sobngwi

**Affiliations:** 1 Institute of Health and Society, Newcastle University, United Kingdom; 2 Catholic University of Central Africa, Cameroon; 3 Department of Endocrinology, University Hospital, Guinea

## Abstract

**Purpose:**

The prevalence of diabetes mellitus is increasing especially in low- and middle-income countries in which 75% of the world’s diabetic population reside. The macro- and microvascular complications of diabetes such as diabetic retinopathy are also set to increase in these populations.

The relationship between depression and glycaemic control has been established in high-income countries, but evidence from low- and middle-income countries is scarce. This research aimed to determine an association between depression and glycaemic control and record the prevalence of diabetic retinopathy in a diabetic population in Cameroon.

**Methods:**

Analysis of cross-sectional data from the ‘Improving access to HbA1c measurements in sub-Saharan Africa’ study was used. Primary data were collected from six diabetic care facilities in Yaoundé, Cameroon. Participants were aged ≥ 18 years with at least a 6-month history of diabetes. Depression was assessed using the Centre for Epidemiological Studies Depression Scale (CES-D). A CES-D score ≥ 16 was used to identify the presence of clinically significant depressive symptoms. Data on glycaemic control were measured using HbA1c measurements at baseline. The presence of diabetic retinopathy was established through ophthalmoscopy and angiography using the Early Treatment Diabetic Retinopathy Study classification.

**Results:**

A total of 261 participants were included in the study, and information on depressive symptoms at baseline (CES-D score) were available for 240 participants. The results of the data analysis found that 60% of the study participants had clinically significant depressive symptoms (CES-D > 16). A weak non-significant positive correlation was found between CES-D score and HbA1c level (*p* = 0.46, *r* = 0.05) using the Pearson’s correlation co-efficient. Gender and attendance to a patient support group were significantly associated with the presence of clinically significant depressive symptoms. Poor glycaemic control (HbA1c > 7%) was found in 72.8% of the population. Educational level and insulin use were significantly associated with glycaemic control.

The prevalence of diabetic retinopathy was 27.2% (23.4% non-proliferative, 2.5% pre-proliferative and 3.2% proliferative), and the prevalence of diabetic maculopathy was 10.0%.

**Conclusion:**

The study found that a large proportion of diabetic patients may be experiencing depressive symptoms for which they are currently not receiving treatment or support. We also found a large proportion to have poor glycaemic control that is known to worsen the vascular complications of diabetes. In light of the increasing epidemic of type 2 diabetes in sub-Saharan Africa, it is important that the recognition of depressive symptoms becomes integrated into future healthcare policies in the nations of sub-Saharan Africa. This research suggests that individuals experiencing depressive symptoms may be more likely to engage in patient support groups. These groups can be beneficial in providing patients with diabetes valuable information, which could lead to better glycaemic control.

## Introduction

Diabetes mellitus is currently one of the most important public health issues of the 21st century affecting an estimated 347 million people worldwide with low- and middle-income countries currently being home to 75% of the world’s diabetic population.^1^ Sub-Saharan Africa is currently facing an epidemic of type 2 diabetes mellitus, thought to have arisen because of rapid urbanisation, an increasingly ageing population and major changes in lifestyle.^2,3^

Depression is the leading cause of disability worldwide and is a major contributor to the global burden of disease, affecting an estimated 350 million people.^4^ Research has shown depression to be associated with a high mortality in people with diabetes.^5,6,7,8^ Patients with diabetes are twice as likely to be depressed compared to those without diabetes.^9^ Alongside this, depression can also lead to an increased risk of the vascular complications of diabetes such as diabetic retinopathy, neuropathy and cardiovascular disease.^10^

Diabetic retinopathy is a microvascular complication of diabetes and is the leading cause of blindness in people of working age in Europe and North America.^11^ In sub-Saharan Africa, the prevalence of retinopathy in patients with diabetes varies across the continent ranging from 16 in some regions to 55% in other regions.^2^ Estimates of prevalence vary according to duration, clinical site and glycaemic control. The contribution to overall blindness in sub-Saharan Africa is unknown, but studies suggest that the proportional contribution may be increasing.^2^ Studies to date involving multiethnic populations living in sub-Saharan Africa have found that visual loss relating to diabetic retinopathy is more likely in populations of African origin compared to those of other ethnic minorities living in sub-Saharan Africa.^12^

Literature on depression in people residing in low- and middle-income countries is limited but growing evidence demonstrates the negative impact of poverty and mental health illness on people living in these regions.^13^ Much of the research surrounding depression and type 2 diabetes that has been conducted in low- and middle-income countries have primarily been conducted in middle-income economies such as India, China, Mexico and Brazil.^14^

Over the past 50 years, there has been an increased awareness of depression in sub-Saharan Africa.^15^ Prior to this, depression was assumed to be non-existent in Africa, and even today, the prevalence may still be underestimated.^16,17^ However, information about depressive symptoms in individuals living with chronic illnesses in sub-Saharan Africa is still scarce.^18^ Despite this, research from low-income economies such as Bangladesh and Guinea suggests that anxiety and depressive symptoms are common in patients living with diabetes.^19,20^

The aim of this research was to analyse the association between depressive symptoms and glycaemic control and record the prevalence of diabetic retinopathy in a clinical sample of patients with diabetes from the ‘Improving access to HbA1c measurement in sub-Saharan Africa’ project.^21^

## Materials and methods

Data were collected from a multicentre hospital cohort involving six diabetic care facilities in Yaoundé, Cameroon. This study was performed as secondary data analysis of the ‘Improving access to HbA1c measurement in sub-Saharan Africa’ study.^22^ The *empirical study* was an interventional study aiming to improve access to HbA1c measurements in sub-Saharan Africa. The empirical research was conducted from 01 November 2008 to 30 April 2012 in Guinea and Cameroon involving a total of 410 diabetic adult patients with both type 1 and type 2 diabetes mellitus and had a 97% response rate.

Secondary data were analysed from a total of 261 of the 410 participants who had HbA1c scores recorded at baseline. The study included participants aged 18 years and older who had at least a 6-month documented history of diabetes. Patients who had been diagnosed for less than 6 months and those with other acute medical or metabolic conditions were excluded from the study.

Primary data were collected through a semistructured questionnaire. The questionnaire was administered by either a nurse or another healthcare professional and contained anonymised information on HbA1c control, socio-demographic characteristics and diabetes-related characteristics and risk factors for diabetes. Data on the presence of diabetic retinopathy and depressive symptoms at baseline were also collected.

The socio-demographic data included age, gender, employment status, marital status and educational level. Educational level was divided into those who had achieved primary education or less (including those with no formal education) and those who had achieved secondary education and higher. Diabetes-related characteristics such as hypertension, obesity, insulin use and glycaemic control were included. Involvement in physical activity and patient support groups were also included. Obesity was measured according to the World Health Organisation classification for adult obesity with a body mass index (BMI) > 30 kg/m^2^ used to define obesity. Information on glycaemic control was obtained through HbA1c measurements. This analysis used anonymised information about HbA1c readings taken in a clinic by a trained physician at baseline.

The presence of diabetic retinopathy was detected through the use of a clinical eye examination, fundoscopy and fluorescein angiography. All cases of retinopathy were detected by an ophthalmologist using retinal photographs. Diabetic retinopathy was classified as absent, non-proliferative, pre-proliferative and proliferative according to the Early Treatment Diabetic Retinopathy Study classification.^23^ The presence of diabetic maculopathy was diagnosed through fundal examination.

Information on depressive symptoms at baseline was assessed through the use of the Centre for Epidemiological Studies Depression Scale (CES-D). The CES-D scale was created by Laurie Radloff in 1977 as a self-administered questionnaire to assess current levels of depressive symptoms in the general population.^24^ Questions for the scale are drawn from other inventories including the Beck Depression Inventory, Zung Self-Rating depression Scale and the Raskin Scale. However, the CES-D scale differs from previous depression scales because of its focus on identifying depressive symptomology in the general population rather than diagnosing depression in a clinical setting.^24^

The CES-D scale was originally designed to screen for depressive symptomology in North American populations; however, it has since been used widely in a variety of sub-Saharan nations such as Uganda, Zambia, Nigeria and Senegal and validated in South Africa, Zambia, Uganda and Nigeria.^25,26,27,28,29^ The 20-item questionnaire centres on the major clinical symptoms of depression. Answers are given on a score of 0–3 relating to the frequency of symptoms experienced during the past week. The possible scores range from 0 to 60 with high scores indicating more depressive symptoms. A recommended score of greater than 16 is usually recommended to suggest the presence of clinically meaningful depressive symptoms. In this study, individuals with CES-D scores > 16 were identified as having clinically significant depressive symptoms.

### Statistical analysis

The original data were obtained within the SPSS statistics 19 package.^30^ Analysis was made using the Minitab 16 and Epi Info™ 7 statistical software.^31,32^ Categorical variables were analysed using chi-squared test or Fisher’s exact tests if the expected cell counts were less than 5. Assessment for effect modification and confounding was conducted.

## Ethical considerations

The National Ethics Committee in Cameroon approved the study. Participants involved in the study gave written informed consent to voluntarily participate in the study and permission to use information for the purpose of secondary data analysis. We certify that all applicable institutional and governmental regulations concerning the ethical use of human volunteers were followed during this research. This research adhered to the Declaration of Helsinki and was reviewed by the Institutional Review Board at Newcastle University.

## Results

A total of 261 individuals were included in the secondary analysis for which HbA1c was recorded at baseline. A total of 240 of these participants had depressive symptoms (CES-D scores) recorded at baseline. The general characteristics included in the study are presented in Table 1. Missing variables were noted and the categorical variables for which there were incomplete data sets were not included in the statistical analysis (see Tables 3 and 4).

**TABLE 1:** Baseline demographics for diabetes-related characteristics in a sample population.

Characteristics (*n* = 261)	Number (*%*)
**Sex**	
Male	114 (43.7)
Female	147 (56.3)
**Marital status**	
Married	170 (65.9)
Single	88 (34.1)
**Educational level**	
Primary or less	112 (42.9)
Secondary or higher	149 (57.1)
**Employment**	
Employed	117 (44.9)
Unemployed	144 (55.1)
**Alcohol consumption**	
Yes	62 (32.1)
No	131(67.9)

The study had a female majority (56.3%) with a mean age was 56 years and a standard deviation (SD) of 12.21. The age data followed an approximately normal distribution, with an age range of 16–85.

Most individuals had secondary education or higher (57.1%), and 42.9% had primary education or less. The majority of the sample population were married (65.9%) and 34.1% were classified as single with just over half of the population (55.1%) identifying as either retired or unemployed.

The majority of participants (92.7%) were reported as having type 2 diabetes mellitus (see Table 2). Just over half of the study participants (57.0%) had diabetes for ≥ 5 years, 31.1% of the population were defined as obese and 57.0% of participants were identified as having hypertension. Insulin use was reported in 36.8% of participants and 72.8% of the study population were identified as having poor glycaemic control (HbA1c level of > 7%). Attendance in a patient support group for people living with diabetes was reported in 20.3%.

**TABLE 2:** Diabetes-related categorical characteristics in the study population.

Diabetes-related characteristics (*n* = 261)	Number (%)
**Type**	
1	16 (6.1)
2	242 (92.7)
Other	3 (1.6)
**Duration**	
< 5 years	110 (43.0)
≥ 5 years	146 (57.0)
**Obesity**	
Yes	79 (31.1)
No	175 (68.9)
**HbA1c (%)**	
Good control (< 7%)	71 (27.2)
Poor control (> 7%)	190 (72.8)
**Hypertension**	
Yes	135 (57.0)
No	102 (43.0)
**Insulin**	
Yes	96 (36.8)
No	165 (63.2)
**Patient support group**	
Yes	53 (20.3)
No	208 (79.7)
**Physical activity**	
Yes	62 (27.7)
No	162 (72.3)
**Retinopathy**	
Absent	115 (72.8)
Non-proliferative	34 (23.4)
Pre-proliferative	4 (2.5)
Proliferative	5 (3.2)
**Maculopathy**	
Yes	13 (10.0)
No	117 (90.0)

### Diabetic retinopathy

Information on diabetic retinopathy were available for 158 (60.0%) of the 261 participants included in this study. Diabetic retinopathy was detected in approximately 25.0% of the subset for which data were available. The majority of cases were non-proliferative (21.5%) with smaller proportions at the pre-proliferative (2.5%) or proliferative (3.1%) stage. The presence of maculopathy was detected in 10.0% of the population (see Table 2).

### Depressive symptomology

Of the 261 participants studied, CES-D scores were available for 92.0% (240) of the population, with data missing for 23 people. For this reason, statistical analysis was only performed with baseline characteristics which had complete data sets for both CES-D score and HbA1c level (see Table 3).

**TABLE 3:** Baseline characteristics of study population analysed against Centre for Epidemiological Studies Depression Scale (CES-D) score.

Categorical grouping variables	CES-D score		Proportion of individuals reporting depressive symptoms (CES-D > 16)			
	Total (*N* = 240)	Mean (s.d.)	Yes (%)	No (%)	95% CI	*p*
**Sex**						
Female	136	20.2 (7.7)	94 (69.1)	42 (30.9)	−0.32 to −0.07	< 0.01
Male	104	17.1 (7.7)	51 (49.0)	53 (51.0)		
**Educational level**						
Primary or less	100	19.7 (7.7)	64 (64.0)	36 (36.0)	−0.19 to 0.06	0.33
Secondary or more	140	18.3 (8.0)	81 (57.9)	59 (42.1)		
**Employment status**						
Employed	106	19.2 (8.5)	61 (57.5)	45 (42.5)	−0.07 to 0.17	0.42
Unemployed	134	18.6 (7.3)	84 (62.7)	50 (37.3)		
**Insulin use**						
Yes	85	20.1 (8.5)	52 (61.2)	33 (38.8)	−0.11 to 0.14	0.86
No	155	18.2 (7.2)	93 (60.0)	62 (40.0)		
**Patient support group**						
Yes	50	19.8 (7.5)	38 (76.0)	12 (24.0)	0.06 to 0.33	< 0.01
No	190	18.6 (7.9)	107 (56.3)	83 (43.7)		
**Glycaemic control**						
Poor (HbA1c > 7%)	176	19.0 (7.9)	108 (61.4)	68 (38.6)	−0.10 to 0.18	0.62
Good (HbA1c ≤ 7%)	64	18.4 (7.6)	37 (57.5)	27 (42.4)		

CI, Confidence interval; s.d., standard deviation.

A large proportion of participants had borderline depressive symptoms with CES-D scores around 15 and 16 at baseline, with scores ranging from 1 to 49 (out of a minimum score of 0 and a maximum score of 60).

Statistical significance was found between reporting of clinically significant depressive symptoms (CES-D score > 16), gender and involvement in a patient support group for diabetes.

The mean CES-D score for men was 17, compared to mean CES-D score of 20 for women. More women (69.1%) reported significant depressive symptoms compared to men (49.0%, chi-squared *p* < 0.01). Individuals who had a primary education or less reported more clinically significant depressive symptoms (64.0%) compared to those with secondary education or more (57.9%, *p* = 0.33). Those who were unemployed reported more depressive symptoms (62.7%) compared to those who were employed (57.5%, *p* = 0.42). Individuals who use insulin also reported more depressive symptoms (61.2%) compared to those who do not use insulin (60.0%, *p* = 0.86). Individuals involved in a patient support group were noted to have more clinically depressive symptoms (76.0%) compared to those not involved in a patient support group (56.3%, *p* < 0.01).

Individuals with poor glycaemic control, HbA1c > 7%, were found to have more depressive symptoms (61.4%) compared to those with good glycaemic control (HbA1c ≤ 7%) (57.5%). A weak positive correlation was found between CES-D score and HbA1c level at baseline, but this was found not to be statistically significant (*r* = 0.05, *p* = 0.46).

### Glycaemic control

The majority of participants in the study (72.8%) were identified as having poor glycaemic control (HbA1c > 7%) at baseline. The median HbA1c score was slightly lower in women (8.0%) compared to men (9.0%). A significant association was found between educational level, insulin use and poor glycaemic control. A greater proportion of people with a higher educational level (12.0%) had poor glycaemic control compared to those with less education (chi-squared *p* = 0.04). A higher proportion (20.0%) of individuals on insulin had poor glycaemic control compared to those not using insulin (chi-squared *p* < 0.01). Men had worse glycaemic control (77.2%) compared to women (69.4%, *p* = 0.15). Those who were employed were identified as having poorer glycaemic control (77.8%) compared to those who were unemployed (68.7%, *p* = 0.10) and those who did not attend a patient support group had poorer glycaemic control (73.6%), compared to those who did attend a patient support group (69.8%, *p* = 0.09) (see Table 4).

**TABLE 4:** Baseline characteristics of study population analysed against glycaemic control.

Categorical grouping variables	Total (*N* = 261)	Good control HbA1c ≤ 7% *N* (%)	Poor control HbA1c > 7% *N* (%)	95% CI	*p*
**Sex**					
Female	147	45 (30.6)	102 (69.4)		
Male	114	26 (22.8)	88 (77.2)	−0.33 to 0.19	0.15
**Educational level**					
Primary or less	112	38 (33.9)	74 (66.1)		
Secondary or more	149	33 (22.1)	116 (77.9)	< -0.01 to 0.22	0.04
**Employment status**					
Employed	117	26 (22.2)	91 (77.8)		
Unemployed	144	45 (31.3)	99 (68.7)	−0.02 to 0.20	0.10
**Insulin use**					
Yes	96	14 (14.6)	82 (85.4)		
No	165	57 (34.5)	108 (65.5)	0.09 to 0.30	< 0.01
**Patient support group**					
Yes	53	16 (30.2)	37 (69.8)		
No	208	55 (26.4)	153 (73.6)	−0.21 to 0.02	0.09

CI, Confidence interval.

## Discussion

The results of the secondary data analysis found that over 60.0% of study participants reported clinically significant depressive symptoms (CES-D > 16) and over 70.0% of participants were reported as having poor glycaemic control at baseline. The study participants were not assessed for a criterion-based mood episode, but it is likely that at least some of these participants would be suffering from a mood episode.

The prevalence of depressive symptoms in this study was higher than that seen in diabetic patients in other low- and middle-income countries such as Jordan (19.7%), Thailand (~25%), India (16.9% – 43.4%) and Guinea (34.4%).^18,33,34,35,36,37^ However, many of these studies differ in the scale and score used to identify depression and the presence of depressive symptoms.

The presence of clinically significant depressive symptoms was found to have a significant association with gender. Women reported higher levels of clinically significant depressive symptoms compared to men. This is similar to results found in other studies surrounding depression in patients with diabetes.^35,38,39,40,41^ Our analysis suggests a significant association between depression and having a lower educational level and being unemployed; however, this was not statistically significant as has been demonstrated in other research.^35,42,43,44,45^

Our study found a weak positive correlation between CES-D score and glycaemic control that was not statistically significant. However, research from various countries suggests a significant association.^36,37,38,41,42,45,46^ Despite much research, the exact direction of the relationship is as yet unknown.^47^ Some studies suggest that depression may be a consequence of living with diabetes mellitus because of factors such as the burden of chronic disease or the biochemical changes that occur in diabetes.^48^ Other studies have proposed that depressive symptoms may be a risk factor for developing diabetes mellitus because of a decline in health maintenance behaviours or biochemical changes associated with depression.^49^

The results of the study also found that individuals who were involved in a patient group had higher levels of clinically depressive symptoms compared to those who were not. These individuals were also found to have better glycaemic control, although this was not significant. This research suggests that individuals experiencing depressive symptoms may be more likely to engage in patient support groups. These groups can be beneficial in providing patients with valuable information, which could lead to better glycaemic control.

### Study strengths

This study is one of the first to date to assess the relationship between depression and glycaemic control in Cameroon. Data were obtained from a diverse range of participants involving six diabetic care populations across Cameroon from the ‘Improving access to HbA1c measurement in sub-Saharan Africa’ study. This makes the data more representative of the population in Cameroon.

### Limitations

A limitation to this study is that a self-report measure was used to assess depressive symptomology. This could explain the higher rates of depressive symptoms in the population as self-report measures often lead to higher rates of depression compared to methods using formal psychiatric interviews.^50^ In this study, CES-D scored > 16 were used to demonstrate clinically significant depressive symptoms. However, some studies have used recommended cut-off points of 16–23 as having ‘distressed symptoms’ and > 23 as being ‘depressed’.^25^ CES-D scores using cut-off points between 21 and 23 have been shown to have a sensitivity and specificity greater than 85%.^51,46^

The CES-D scale has also not yet been validated for use in Cameroon but has been widely used and validated for research in patient populations in various sub-Saharan African nations.

## Conclusion

The established increase in diabetes mellitus in low- and middle-income countries indicates the need for further research. In particular, the association between depressive symptoms and glycaemic control should be explored to allow close monitoring of HbA1c in individuals most at risk. The finding from this piece of research highlights the need for increased monitoring of HbA1c levels and detection of depressive symptoms, which might be consequent to a mood disorder. This should be achieved through appropriate and valid assessment by healthcare professionals, particularly in high-risk groups such as women, the unemployed, those with lower levels of education and those using insulin. It also suggests that patient support groups in low- and middle-income countries could help those most at risk of depressive symptoms and encourage education on how to maintain better glycaemic control.
